# Road Map for Nanocrystal Based Infrared Photodetectors

**DOI:** 10.3389/fchem.2018.00575

**Published:** 2018-11-28

**Authors:** Clément Livache, Bertille Martinez, Nicolas Goubet, Julien Ramade, Emmanuel Lhuillier

**Affiliations:** Sorbonne Université, CNRS, Institut des NanoSciences de Paris, INSP, Paris, France

**Keywords:** infrared, nanocrystals, photodetection, device, interband, intraband

## Abstract

Infrared (IR) sensors based on epitaxially grown semiconductors face two main challenges which are their prohibitive cost and the difficulty to rise the operating temperature. The quest for alternative technologies which will tackle these two difficulties requires the development of new IR active materials. Over the past decade, significant progresses have been achieved. In this perspective, we summarize the current state of the art relative to nanocrystal based IR sensing and stress the main materials, devices and industrial challenges which will have to be addressed over the 5 next years.

## Introduction

Over the recent years, colloidal quantum dots (CQDs) have reached a first mass market application with their use as light sources for displays. This application brought even more interest for CQDs both at the academic and industrial levels. Among emerging applications, infrared (IR) photodetection (Kershaw et al., 2013; Lhuillier and Guyot-Sionnest, 2017) is a field where colloidal materials have a strong potential to bring cost disruption, especially because organic semiconductors, often seen as the low-cost alternative to conventional semiconductors, are ineffective in this range of wavelengths.

IR detection currently relies on two types of sensors. Quantum detectors are based on photon-absorbing semiconductor materials. These can either be narrow band gap semiconductors (InGaAs in the short-wave IR, InSb in the mid-wave and HgCdTe for both mid- and long-wave IR) or semiconductor heterostructures (GaAs/AlGaAs, used in Quantum Well Infrared Photodetector or QWIP, and InAs/GaSb in type II superlattices). These technologies are mature, present high performances (i.e., high quantum efficiency, relatively low dark current, high uniformity, fast time response), but suffer from an excessive cost (a typical IR camera costs 30 k−100 k€) and a low operating temperature. As a result, access to this type of technologies remains restricted to defense and scientific applications (mostly astronomy).

The second class of IR detectors is thermal detectors, sensitive to energy flux rather than photon flux. Materials used for this kind of detectors see one of their physical property (typically, their electrical resistance) changing upon absorption of IR radiation. Typical technologies are bolometers and pyrometers. Their operation principles make them intrinsically slower than quantum devices, and they present lower detectivity (signal to noise ratio) than their quantum detector counterpart. On the other hand, they can be operated at room temperature and their cost is significantly lower, ranging from 100 € to few k€ per focal plane array (FPA) and 1 to 10 k€ for camera.

To bring IR detection to a mass market level, a technology combining both the performances of quantum detector and the low cost of thermal detector needs to emerge. CQDs appear as promising candidates to reach this goal. Beyond their tunable absorption from the near IR to the THz range (Goubet et al., 2018a), several significant proofs of concepts such as mid-IR photoconduction (Keuleyan et al., 2011), background limited photodiode (Guyot-Sionnest and Roberts, 2015) and investigation of stability issues (Jagtap et al., 2018a) have brought CQDs to a technological readiness level (TRL) above 3 which is critical for the industry to start considering an emerging technology.

The focus of this paper is intentionally limited to IR detection, which means that we have excluded from the scope of this review any solar cell application (Sargent, 2012). In this perspective, we propose a road map of the main challenges that have to be addressed by the community in order to transfer the IR CQD technology to the industrial level.

## Discussion

### Basic of IR detection using nanocrystals as active material

**Figure 1:**
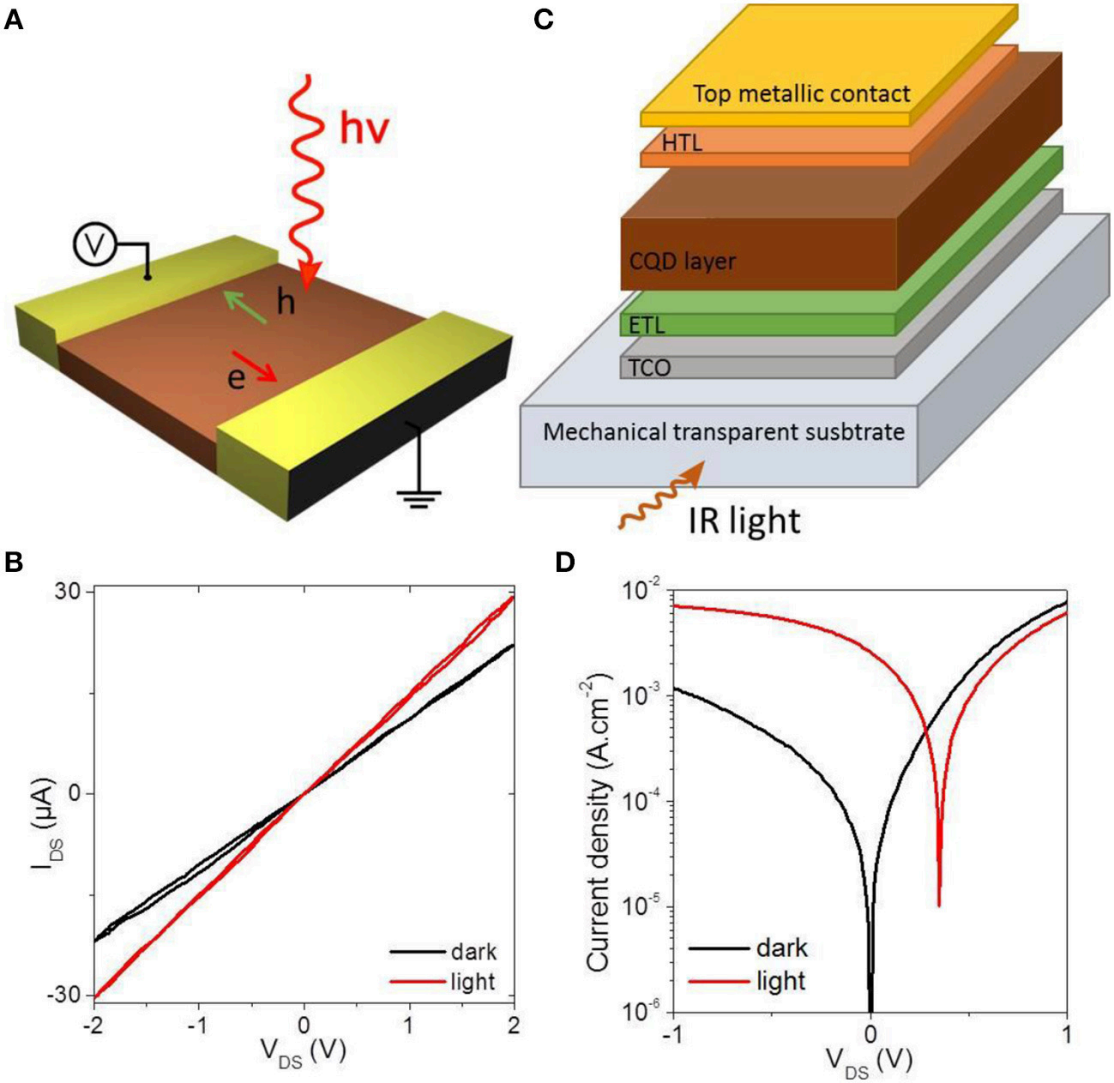
**(A)** Scheme of a photoconductive device in planar geometry. **(B)** I-V curve of a photoconductive device under dark condition and under illumination. **(C)** Scheme of a photodiode in vertical geometry. TCO, ETL and HTL stands respectively, for transparent conductive oxide, electron transport layer and hole transport layer. **(D)** I-V curve of a photodiode under dark condition and under illumination.

To start, we would like to discuss the basics of the transformation of a colloidal nanocrystal solution into an IR sensor. Two type of geometries have been explored: planar and vertical geometry, see Figures 1A,C. The planar geometry is certainly the easiest to implement, because this geometry is far less sensitive to the film quality (i.e., film roughness and cracks do not lead to electrical shorts in the device). The success of this geometry also relates to the possibility to add a gate for the design of field effect (photo)transistor (Talapin and Murray, 2005). Typically, electrodes are prepared on a conventional substrate (Si/SiO_2_ typically). Interdigitated electrodes have been widely used as a strategy to enhance the current magnitude. The film of CQD is deposited on this substrate using methods such as spin coating, dropcasting, dip coating or spray coating (Cryer and Halpert, 2018). As is, the film of nanocrystal is insulating and a ligand exchange step is necessary to increase the CQD electronic coupling and achieve photoconduction. IR exposition is obtained by top side illumination. Typical I-V curve from such planar photoconductive device is shown in Figure 1B. The photosignal relates to the modulation of the I-V curve slope. Field effect transistor configuration (Lhuillier et al., 2014b) is interesting to tune the majority carrier current and possibly enhance the signal to noise ratio. The gating is typically obtained through the use of the dielectric layer from the substrate or through the deposition of a top side dielectric (Chung et al., 2012) or electrolyte (Lhuillier et al., 2014b).

The second type of detector geometry that has been widely explored is based on a vertical geometry. The typical stack of layers relies on a transparent substrate (glass in the near infrared) on top of which a transparent conductive layer is deposited. ITO (indium tin oxide) and FTO (fluoride doped tin oxide) are the most used material. An electron transport layer (ETL) is then deposited: the most used material for CQD based device are inorganic layers made of ZnO or TiO_2_. This layer generally needs to be annealed at high temperature, which makes that it is highly desirable to process it as a bottom layer (i.e., before the CQD deposition). On the top of the ETL, the CQD layer is deposited. The typical thickness of this layer range from 200 to 400 nm. This value is a trade-off: thicker layer might be desirable to absorb more light since only 10–30 % of the light are absorbed in those conditions (Cademartiri et al., 2006; Hens and Moreels, 2012). However, thicker layers are difficult to build due to the multiplication of deposition and ligand exchange steps. Moreover, the short transport diffusion length makes that photocarriers might not be collected in thicker layers. On the top of the QD layer, a hole transport layer can be deposited. MoO_3_ has been intensively used in the case of CQD (Gao et al., 2011; Chuang et al., 2014). Finally, a top metallic contact is deposited. There are many possible alternative configurations to the one described above with inverted geometry, as the combination of a n and p type layer (Chuang et al., 2014) or graded band gap configuration (Kramer et al., 2011) to funnel charges to the contacts. In this vertical configuration, illumination is made through the substrate, bottom contact and ETL. A typical IV curve of such photodiode is shown in Figure 1D. The key advantage of this configuration is to be able to operate the device close to zero bias to reduce the dark current, by taking advantage of the built-in electric field of the diode.

**Figure 2:**
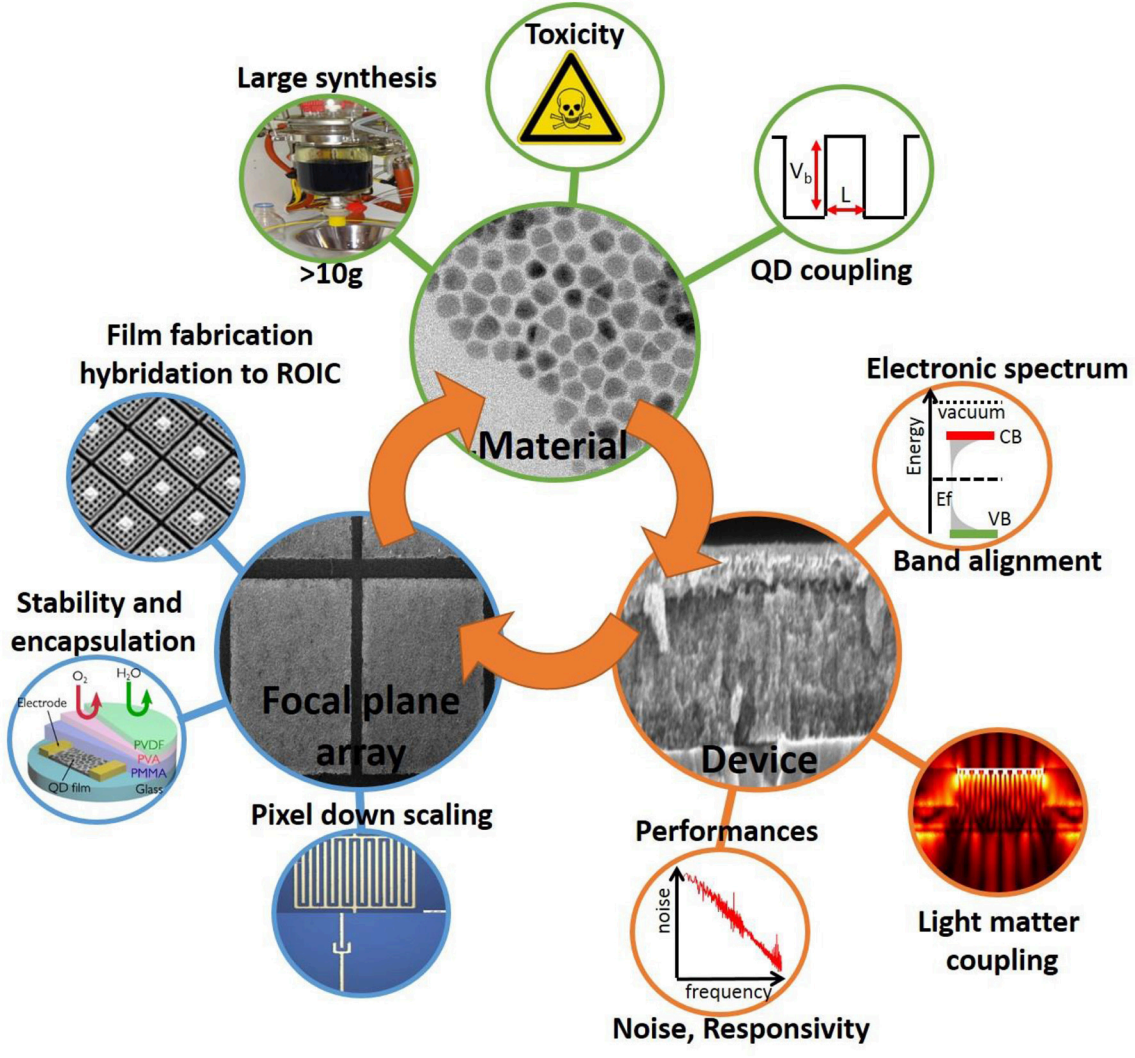
Illustration of the main challenges that need to be addressed to bring the field of IR nanocrystals to a mature level for thermal imaging.

Now that the basic of CQD based IR detector design being established, it is of utmost importance to remind the main figures of merit relative to IR sensing (Rosencher, 2002). Responsivity (in A/W) is the first figure of merit which translates the ability of the active layer to transform a light signal into an electrical signal. This quantity directly relates to the external quantum efficiency (efficiency to convert incident photons into electrical current) and to internal quantum efficiency (efficiency to convert absorbed photons into electrical current, in other word the external quantum efficiency normalized by the device absorption). One of the key specificities of IR is the limited signal-to-noise ratio. Indeed, because of the narrow energy transition involved in the IR, thermal activation competes with photon activation of the carriers. This results in a dark current which can be a significant fraction of the total current. The relevant contribution of the dark current to noise is its spectral distribution (in A.Hz^−1/2^), Hence, the quantity involved in the ultimate figure of merit of an IR detector is the detectivity (signal to noise ratio expressed in cm.Hz^1/2^.W^−1^ or Jones). Currently, all convincing reports relative to the measurement of noise in nanocrystal arrays have led to *1/f* noise as the prevailing contribution (Lai et al., 2014; Liu et al., 2014; De Iacovo et al., 2017). It is a very common habit to observe detectivity value reported assuming that noise is shot noise limited (mostly because there is an analytical expression for shot noise and none for *1/f* noise), however this leads to a huge overestimation of the device detectivity. Finally, another important figure of merit which differentiates detectors from solar cells is the device time response. To take full advantage of photon detectors, faster time responses that the ones reported for thermal detectors (≈10 ms) are highly desirable. In the following we discuss state of the art results and expected performance targets for SWIR, MWIR, and LWIR range of wavelengths. We will now discuss the main challenges to address in order to bring the CQD technology to the industrial level. We have sorted those in three main categories: (i) material, (ii) device and (iii) camera integration challenges, see Figure 2.

### Material challenges

#### IR absorption: interband vs. intraband transitions

Among all criteria to build an IR detector, design IR absorption appears as the first challenge to tackle. Three wavelength bands appear promising for applications: short-, mid- and long-wave infrared.

**Figure 3:**
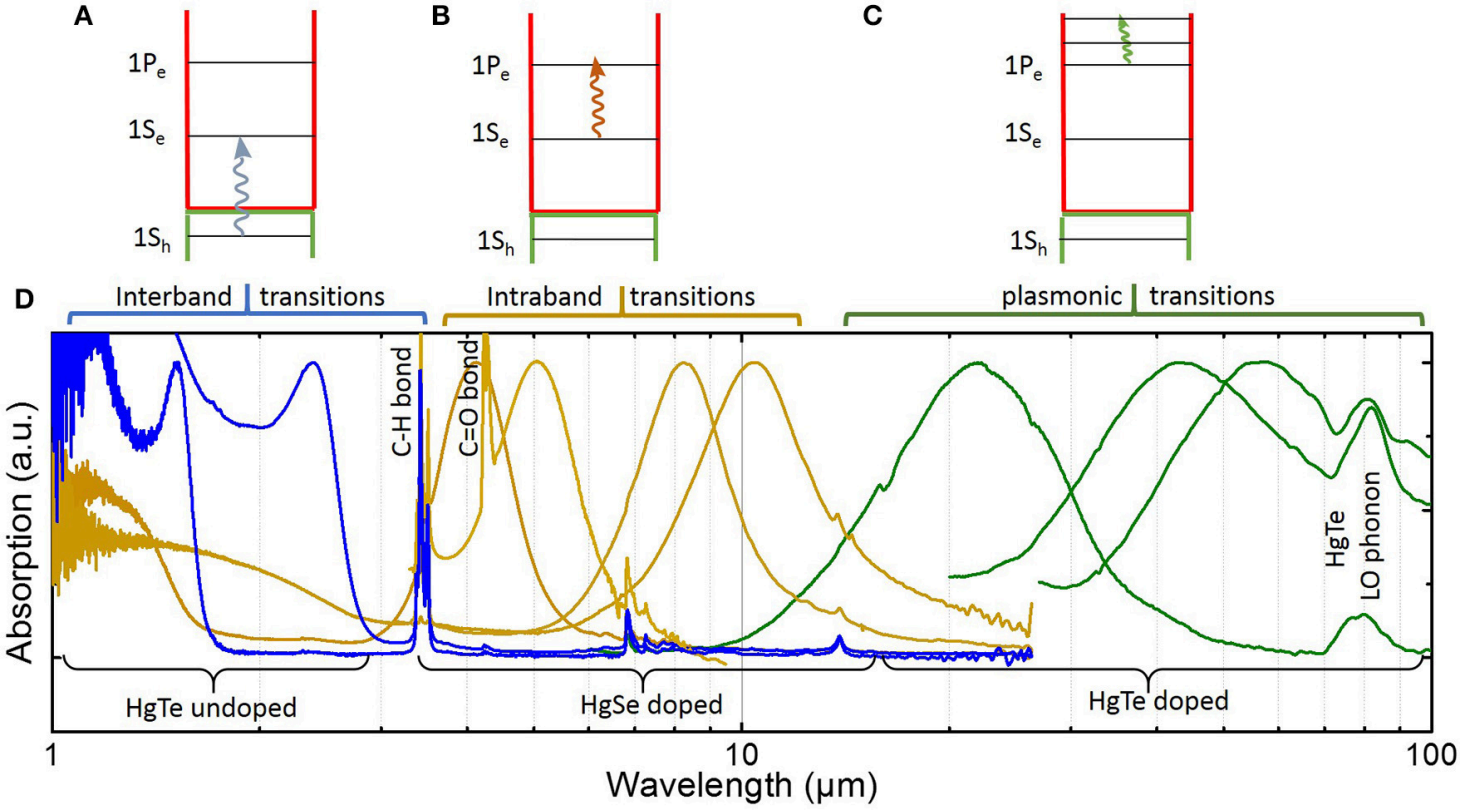
**(A–C)** are respectively, the scheme for interband, intraband, and plasmonic transitions in nanocrystals. **(D)** Absorption spectra for mercury chalcogenide (HgSe and HgTe) nanocrystals of various sizes.

Short-Wave Infrared (SWIR) extends from 800 nm to 1.7 μm, and up to 2.5 μm for extended SWIR. In this range, the objective for CQD-based detectors is to offer an alternative to InGaAs. As stated earlier, these technologies offer top-level performances. However, their cost, without being prohibitive, remains far above comparable technologies in the visible range. Moreover, the perspectives of cost disruption are limited for such a mature technology. In this range of wavelengths, applications are typically active imaging, night glow assisted imaging and tissues imaging. Among possible colloidal materials to be used in this range of wavelengths, two materials have reached a large enough maturity: lead chalcogenides (Sargent, 2008) (PbS and PbSe, mostly) and HgTe (Kovalenko et al., 2006; Keuleyan et al., 2011; Green and Mirzai, 2018). In those materials, IR absorption is obtained through interband transitions, see Figure 3A.

In the Mid-Wave Infrared (MWIR: 3–5 μm), blackbody emission of room-temperature objects starts to prevail over reflection of other light sources, hence opening the field of thermal imaging. In this range, HgTe is by far the most investigated material (Kovalenko et al., 2006; Keuleyan et al., 2011; Tang et al., 2016) thanks to its tunable interband IR transition, see Figure 3D. Another strategy to achieve low-energy transition in the MWIR is to use intraband transitions (see Figure 3B; Deng et al., 2014; Jagtap et al., 2018b; Kim et al., 2018). In this case, the transition occurs in the first levels of the conduction band, hence doped semiconductors are necessary. Again, mercury chalcogenides are the most investigated materials for photodetection thanks to self-doping (Deng et al., 2014; Jagtap et al., 2018b; Kim et al., 2018).

Long-Wave Infrared (LWIR: 5–30 μm) is the optimal range to perform thermal imaging of room-temperature objects since their blackbody emission maximum lies around 10 μm. Addressing such low energy with interband transitions is extremely challenging since the confinement energy needs to be so small that the required size of nanocrystals becomes incompatible with colloidal stability or monodispersity. Intraband (Park et al., 2018) and plasmonic transitions (Luther et al., 2011; Agrawal et al., 2017; Coughlan et al., 2017; Askari et al., 2018; Liu et al., 2018) (achieved at a higher doping level) in doped nanocrystals, see Figure 3C, are interesting for two reasons: (i) addressing long wavelengths from the MWIR to the THz range while keeping the colloidal stability of the material and (ii) because doping of nanocrystals might not be limited to toxic material.

#### Surface chemistry

Beyond the nanocrystal synthesis, the control of surface chemistry is a critical step toward the design of photoconductive thin films. Initial long capping ligands, which ensure the nanometer size growth and preserve the colloidal stability, need to be stripped from the surface to increase the inter-CQD coupling (i.e., to reduce the inter CQD tunnel barrier) and obtain reasonable carrier mobility. For most of the reported devices the ligand exchange remains based on a solid state ligand exchange (i.e., performed on the film), typically using ethanedithiol as capping ligand (Lhuillier et al., 2013). This undoubtedly limits the carrier mobility in the 10^−3^ cm^2^V^−1^s^−1^ range and likely the associated photoresponse. Introduction of inorganic ligands such as As_2_S_3_ appears as an interesting path to obtain higher film mobility (Lhuillier et al., 2013; Yakunin et al., 2014; Tang et al., 2016; Cryer and Halpert, 2018). It is nevertheless as important to boost mobility as preserving a good CQD surface passivation, especially for the design of photodiode, and more work needs to be done in this direction. In the case of ink preparation, CQD ends up being ligand-exchanged and suspended in polar solvent with high boiling point, raising some questions relative to the film preparation. Indeed, most of the devices are currently prepared using dropcasting (Tang et al., 2018) or spin coating. The latter method is difficult to implement using high boiling point solvent, in addition to a dramatically low efficiency (i.e., 90% of the material is wasted). Among alternative methods, dip coating (Chernomordik et al., 2017) and spray coating (Chen et al., 2013; Wang et al., 2015; Cryer and Halpert, 2018) have also been reported. The choice for a given method also impacts the preparation of the QD ink: low concentrations are used for dropcasting (10 mg/mL), while higher concentrations (50 mg/mL) are used for spin coating in non-polar solvent and even higher in the case of spin coating from polar solvent. There is probably no perfect method for deposition and each involved team has to pay the price of time-consuming optimization for this step.

Another main challenge relative to the use of mercury chalcogenides relates to the softness of the material. As a result, any annealing step (to boost the mobility or as part of a lithography process) leads to sintering of the nanocrystal film. This induces an increase of the CQD effective radius which broadens the absorption cut-off and, even worse, dramatically increases the dark current (through a reduction of the effective band gap). Thus, core-shell objects with an external material which is able to sustain temperatures around 160°C (i.e., typical baking temperature of lithography resist such as polymethyl methacrylate) without aggregation will be of utmost interest. Introduction of core-shell structure may also lead to longer living photocarriers which is highly desirable for photodetection. A first study has been done in this direction by coupling HgTe and HgSe materials in heterostructured nanocrystals (Goubet et al., 2018b). However, growing such shell remains quite challenging because HgX compounds are grown at low temperature (100°C typically), while conventional shell materials are synthetized at high temperature (200°C and more). Currently all reported HgX based core-shell materials remain based on the room temperature Colloidal-Atomic Layer Deposition (C-ALD) process (Robin et al., 2016; Shen and Guyot-Sionnest, 2016; Sagar et al., 2017), which appears to be suboptimal procedure (Lhuillier et al., 2014a). Shell growth method specific to soft materials will have to be developed.

#### Material toxicity

Material toxicity is probably one of the most difficult issues to address. As stated previously, the field remains driven by lead and mercury chalcogenides, which are actually the same material as the ones used in current IR detection technologies. For sure, the introduction of low toxicity compounds will be a breakthrough in the field. In the visible range of wavelengths, a large effort has been devoted to the synthesis of InP as an alternative to CdSe as light source for display. On the other hand, colloidal narrow band gap III-V materials (InAs Franke et al., 2016; Grigel et al., 2016; Srivastava et al., 2018 and InSb Maurice et al., 2013; Chang et al., 2014) remain poorly mature with a too limited amount of reports and none dedicated to IR detection further than the SWIR. First issue is due to the more covalent character of III-V materials with respect to II-VI materials. As a result, more reactive precursors need to be used which leads to a higher degree of synthesis complexity. Second, the availability of stable pnictogen precursors is decreasing for narrow band gap materials mostly due to their atomic radius increasing.

Narrow band gap interband transitions are more likely to be observed using heavy elements (higher Z value comes with a denser density of states, which are more likely to present a narrow band gap in the vicinity of the Fermi level). However, materials in the bottom part of the periodic table are also toxic. Certainly, a switch toward intraband transition is the promising path to achieve heavy metal free IR detection. This strategy is nevertheless currently limited to the MWIR and LWIR and the level of performance is by far not as good as their interband counterparts. Ag_2_Se, thanks to its spectral vicinity with HgSe has recently generated some interest for MWIR detection (Sahu et al., 2012; Park et al., 2018; Qu et al., 2018), although detection performances remain for now several orders of magnitude below than what have been reported with HgX compounds.

Another class of material that may appears as promising to achieve infrared absorption are strongly-doped semiconductors with plasmonic absorption in the infrared. This is typically the case of copper chalcogenides (Dorfs et al., 2011; Kriegel et al., 2012; Coughlan et al., 2017), oxide nanoparticles (Kanehara et al., 2009; Buonsanti et al., 2011; Della Gaspera et al., 2013; Ghosh et al., 2014; Schimpf et al., 2015; Runnerstrom et al., 2016; Tandon et al., 2017) or doped silicon (Gresback et al., 2014; Zhang et al., 2017). Plasmonic nanoparticles tend to have a much higher cross section (≈10^−13^ cm^−2^/particle) that the one associated with interband or intraband transitions (10^−15^-10^−14^ cm^−2^/particle range). However, their very short living photocarrier (<1 ps) might strongly balance the absorption enhancement. The investigation of (photo)transport properties in those materials needs to be pushed further.

#### Scale-up for production

When it comes to mass market application, a first question to consider is how much material will be necessary. Let's assume that the objective is to provide to every car sold in Europe (≈20 million unit per year) an IR sensor dedicated to night-driving assistance. Typical device will have a 1 cm^2^ size for a thickness of a few hundreds of nm (400 nm for the calculation). Let's assume further a density of 10 for the material with a film filling of 0.64, corresponding to a randomly close-packed film. Moreover, we can account for the poor efficiency of deposition method such as spin coating where 90% of the material is wasted. A single device thus requires 2.6 mg of active material. This means that around 50 kg of HgTe will be necessary to saturate the targeted market. It is worth pointing that this amount is actually quite small and material supply is not an issue. This strongly contrasts with solar cell applications, where much larger devices (m^2^) are necessary and where consequently material supplying (in particular Te, for which supply short fall is expected 10 years from now) and toxicity become critical issues.

Large scale synthesis of quantum dots (Protière et al., 2011) and more particularly mercury chalcogenides at the 10 g scale has already been reported (Lhuillier et al., 2016). Thus, to reach an annual production of 50 kg, batches reaching a few 100 of g will be needed. This is probably a step where small and medium size companies producing nanocrystals should be involved to take advantage of the know-how developed for wide band gap materials (i.e., CdSe and InP).

Regarding the material production cost, it was recently evaluated by (Jean et al., 2018) that PbS CQDs present a fabrication cost in the 10 to 60 $/g range. Thus, even assuming that for HgTe the fabrication cost will be in the upper range of this estimation, the cost of active material per device remains extremely low (0.15 $). We can conclude that contrary to solar cells, the material cost is here not a limiting factor.

### Device challenges

#### Electronic structure and band alignment

As stated earlier, the most promising colloidal materials for IR detection are lead and mercury chalcogenides. Knowledge of the electronic structure of these material remains limited compared to the one reached for silicon and III-V semiconductors, and most electronic structure parameters are known within a limited accuracy, even for the bulk. Once under colloidal form, we add on the top of that quantum confinement, dependence of the electronic spectrum with surface chemistry and surface traps. As a result, the electrical landscape of our active material remains quite blurry. It is consequently difficult to design device with carefully-optimized band alignments and ohmic contacts. A significant effort will have to be done to provide to the community data relative to the electronic structure of the infrared colloidal materials in a quite systematic way (material, size, surface chemistry). First results in this direction have been reported using combination of IR spectroscopy and electrochemistry (Chen and Guyot-Sionnest, 2017) or photoemission measurements (Martinez et al., 2017), but more will be needed.

As critical is the investigation of carrier dynamics in these narrow band gap materials especially for the understanding of current performances limitations. Methods based on time-resolved optical spectroscopy are difficult to transfer in the IR due to an intrinsically low PL efficiency of IR nanocrystals and due to less advanced optical setups in the IR. Development of alternative methods will be required. Again, some preliminary results in this direction have been obtained using time resolved photoluminescence (Keuleyan et al., 2014b), transient absorption (Melnychuk and Guyot-Sionnest, 2018), time resolved photoemission (Spencer et al., 2013; Livache et al., 2017) and transient photocurrent measurements (Gao et al., 2016; Livache et al., 2018; Martinez et al., 2018). There are nevertheless not enough data to depict the full range of dynamics in this material from Auger recombination at short time scale to long-lived traps at long time scale.

Thanks to the understanding of the electronic spectrum, the objective will be the design of new photodiodes, accounting for specificity of IR and colloidal material. While in the SWIR, exploiting the concept developed for solar cell was still a reasonable assumption (Jagtap et al., 2018a), this is no longer the case for longer wavelengths. Alternatives to current electron (mostly ZnO and TiO_2_) and hole transport layers (MoO_3_) will have to be developed. This is even more true to implement the concept of unipolar barrier, which role is to let selectively one carrier flow, while preventing the other carrier to circulate. This concept has been widely used for III-V and II-VI semiconductor IR sensors (White, 1987; Savich et al., 2011, 2013), but remains poorly used in the case or CQD based devices. This might nevertheless be a time-consuming process because the fragility of the IR CQDs will likely require the development of specific carrier transport layer which doping and band alignment will have to be finely tuned and experimentally determined.

#### Device performances

Since the main purpose of this road map is the design of effective IR-sensing devices, it is certainly worth determining which level of performances seems to be a reasonable goal to achieve for colloidal-based IR detectors in each specified range of wavelengths.

##### SWIR

For wavelengths up to 1.7 μm, the key competitor is the InGaAs technology which performance is again very unlikely to be beaten. Thus, CQDs appear promising for (i) cost disruption and (ii) extending the range of wavelengths toward the so-called extended SWIR (2.5 μm cut-off wavelength) (Jia et al., 2018). InGaAs succeeded to achieve extremely low dark current (<20 fA at 20°C for a 15 μm pixel). Because CQD based devices reported so far are not reaching such low dark current densities, operational scenarii with high photon flux seem more appropriate. This typically relates to active imaging (Geyer et al., 2013), flame detection (Iacovo et al., 2017) and biological tissues imaging for which reasonably fast detection is necessary (sub-ms is mandatory and μs is probably a more appropriate target) and already reported (Lhuillier et al., 2013). Also note that in the SWIR, room temperature operation or at least above water freezing point has to be achieved to preserve the low-cost character of the device. Because this range of wavelengths is fairly easy to reach using PbS CQDs and because of the relatively easy device characterization at such wavelengths, very high performance devices (up to kA.W^−1^ for responsivity and detectivity reaching 10^13^ Jones at 1.4 μm) have been achieved (Yakunin et al., 2014). Device demonstration also includes imaging systems (Calvez et al., 2011; Klem et al., 2015).

##### MWIR

In this range, targeted applications are thermal imaging (Tang et al., 2018) and gas sensing (Chen et al., 2017). Current bulk-based imaging devices (typically based on InSb and HgCdTe) have presently no road map for operating temperature above 180°C. This clearly sets a first objective. In the scope of preserving low-cost, it likely suggests that only Peltier cooling should actually be used, bringing the targeted operating temperature around 250 K. Recently reported reduced Auger effect (Melnychuk and Guyot-Sionnest, 2018) in HgTe CQDs compared to their bulk HgCdTe equivalent raises great hope to achieve this goal. In terms of performances, detectivity in the few 10^9^ Jones at room temperature and reaching 10^10^ Jones at 250 K is a clear objective which will bring CQD based technologies above what can be achieved using thermal sensors. Huge progresses in this direction have been reported this year. This includes responsivity above 1 A.W^−1^ (Tang et al., 2018), detectivity above 10^9^ Jones at room temperature (Cryer and Halpert, 2018) and NETD (noise equivalent temperature difference) down to 14 mK (Tang et al., 2018). In the MWIR, the device complexity lags far behind what has been achieved in the SWIR with just a few photodiodes (Guyot-Sionnest and Roberts, 2015; Ackerman et al., 2018) and only one report for focal plane array integration (Buurma et al., 2016).

##### LWIR

When long wavelengths (8 μm and more) start being involved, the number of reported devices quickly drops (Keuleyan et al., 2014a). It consequently becomes difficult to set some objectives of performance. One key difficulty in this range of wavelengths comes from the fact that it is unlikely that non-cryogenic operating temperatures can be achieved. Thus, the cost disruption brought by the CQDs is not as important as in the MWIR. Since the operating temperature of conventional IR camera in the 8–12 μm range is around 90 K, it is likely that operating temperature for CQD based device around 150 K will be desirable, with a detectivity remaining above 10^10^ jones. Preserving a fast response is also key aspect to compete with bolometers. Regarding the THz range, there is currently no available data.

#### Device geometry and emerging strategies

Among current difficulties relative to colloidal materials, one key limitation remains connected to the short carrier diffusion length (50 to 100 nm typically) which is shorter than typical absorption length (close to 10 μm Lhuillier et al., 2012 for a band edge in the MWIR), due to the low carrier mobility. In other words, photogenerated carriers are only collected in the vicinity of the electrodes, while the bulk of the film leads to photogenerated charges that end up being trapped. In this sense, strategies to enhance the light-matter coupling are necessary. The objective of this strategy is to concentrate the incident electromagnetic field on the thin optically absorbing layer of CQDs. Some early results have been reported with the introduction of colloidal gold nanorods (Chen et al., 2014) or of resonators (Yifat et al., 2017; Tang et al., 2018). The development of such plasmonic resonance has also been used to obtain polarized emission and imaging system (Le-Van et al., 2016; Yifat et al., 2017).

Another interesting development that has been reported relates to the design of multicolor detectors. This includes visible and MWIR (Lhuillier et al., 2014b), MWIR/LWIR (Tang et al., 2016) and MWIR/MWIR (Cryer and Halpert, 2018) sensors. However it remains unclear if bicolor technology can be compatible with low fabrication cost.

Finally, it is worth mentioning a strategy which has been explored over the last 5 years to boost the device photoresponse is the coupling of the nanocrystals with a 2D material. Graphene is the first to have been explored. Gigantic responsivities (10^7^ A.W^−1^) have been reported (Konstantatos et al., 2012; Sun et al., 2012) but the concept was unsuccessful because the dark current was even larger than in a conventional film of CQDs. The concept was then revisited by replacing graphene by MoS_2_ to introduce a gap and reduce the dark current. Simultaneously, the absorption was pushed from telecom range to longer wavelengths by replacing the PbS CQDs by HgTe nanocrystals (Huo et al., 2017). This strategy keeps suffering from two main limitations, which are the fast saturation of the optical response (responsivity is only large under zero photon flux) and large memory effects (i.e., long time response).

### Focal plane array

#### Pixel downsizing and coupling to read out circuit

When it comes to building a focal plane array, the pixel size matters. Most of current devices reported in the literature are based on a chip with several individual devices, each of them being typically around 1 mm^2^ area. When such size is far too small in the case of solar cells where m^2^ are required, it is also far too big for use in cameras, where the pixel should be ideally just above the targeted wavelength to improve image quality. Current IR technologies present pixel sizes in the 10 to 50 μm range. These values are actually limited by technology and more specifically by the indium bump hybridization to the read-out circuit, which becomes very complex for size below 10 μm. There is, in this sense, a true opportunity for quantum dots here (Malinowski et al., 2017). Because the active layer can be directly deposited on a CMOS read-out circuit, demonstration of pixel sizes below 10 μm will bring a significant advantage to CQDs. This nevertheless has still to be demonstrated and also raises new questions such as the ability to design large scale homogeneous films while preventing pixel cross-overs. In the hypothesis where optical coupling between pixels will prevent the use of a continuous film of CQD, the film will have to be etched to effectively split the pixels (Lhuillier et al., 2016). In this case, the material will have to sustain a lithography step (i. e., high temperature exposure and exposition to solvent).

#### Stability and encapsulation

Long term stability of CQD based device is a problem which has been mostly swept under the rug. It is conveniently admitted that stability of CQDs is higher than the one of organic materials, however there is a clear lack of data regarding this question. Probably absorption is a much more robust property than photoluminescence, nonetheless lead chalcogenides get oxidized and quickly get a PbO shell, when mercury chalcogenides are also air sensitive (Lhuillier et al., 2013; Jagtap et al., 2018a) even if the exact mechanism remains unclear. Two paths can be followed to address this question: either tuning the surface chemistry to make the CQDs stable in air, or processing the material in air-free conditions followed by the encapsulation of the CQD-based device below a protective layer. Handling of CQDs in glove box has become more or less the regular procedure in the field. Far less work has been devoted to the question of encapsulation. Certainly, concepts from the field of organic electronics and CQD based solar cells (Tan et al., 2017) can be reused. This include deposition of encapsulation polymers such as CYTOP, ALD (atomic layer deposition) deposition of thick alumina layer (Ihly et al., 2011) or nanoparticle shelling (Durmusoglu et al., 2017). Nevertheless, it is critical to consider that current PbX and HgX materials are synthetized at low temperature (<150 and <100°C, respectively) and that processing them at temperatures higher than their growth temperature will undoubtedly lead to a significant sintering and its associated dark current rise. As a result, specific low temperature methods need to be developed. Recently (Jagtap et al., 2018a) have reported the low temperature deposition of a combination of water-proof (PMMA and PVDF) and oxygen-proof (PVA) layers, leading to stability over at least 3 months. This is comparable to the stability obtained for solar cell based on PbS CQDs (Chuang et al., 2014). Longer stability investigation (at least up to 1 year) under realistic operational environment will have to be done to confirm the potential of the technology.

## Conclusion

Thanks to 10 years of intensive research, IR CQDs have undoubtedly reached a maturity level where they can be considered as a possible alternative to historical semiconductors for IR sensing. Main achievements include full tunability of the absorption over the IR range, BLIP photodiode, demonstration of CQD based focal plane array in both SWIR and MWIR range. We have tentatively listed the main technological challenges that still need to be addressed to fully transfer this technology to industry.

## Author contributions

All authors listed have made a substantial, direct and intellectual contribution to the work, and approved it for publication.

### Conflict of interest statement

The authors declare that the research was conducted in the absence of any commercial or financial relationships that could be construed as a potential conflict of interest.

## References

[B1] Ackerman M. M. Tang X. Guyot-Sionnest P. (2018). Fast and sensitive colloidal quantum dot mid-wave infrared photodetectors. ACS Nano 12, 7264–7271. 10.1021/acsnano.8b03425 29975502

[B2] Agrawal A. Johns R. W. Milliron D. J. (2017). Control of localized surface plasmon resonances in metal oxide nanocrystals. Annu. Rev. Mater. Res. 47, 1–31. 10.1146/annurev-matsci-070616-124259

[B3] Askari S. Mariotti D. Stehr J. E. Benedikt J. Keraudy J. Helmersson U. (2018). Low-loss and tunable localized mid-infrared plasmons in nanocrystals of highly degenerate InN. Nano Lett. 18, 5681–5687. 10.1021/acs.nanolett.8b02260 30137994

[B4] Buonsanti R. Llordes A. Aloni S. Helms B. A. Milliron D. J. (2011). Tunable infrared absorption and visible transparency of colloidal aluminum-doped zinc oxide nanocrystals. Nano Lett. 11, 4706–4710. 10.1021/nl203030f 21970407

[B5] Buurma C. Pimpinella R. E. Ciani A. J. Feldman J. S. Grein C. H. Guyot-Sionnest P. (2016). MWIR imaging with low cost colloidal quantum dot films, in Optical Sensing, Imaging, and Photon Counting: Nanostructured Devices and Applications 2016 (San Diego, CA: International Society for Optics and Photonics), 993303 10.1117/12.2239986

[B6] Cademartiri L. Montanari E. Calestani G. Migliori A. Guagliardi A. Ozin G. A. (2006). Size-dependent extinction coefficients of PbS quantum dots. J. Am. Chem. Soc. 128, 10337–10346. 10.1021/ja063166u 16881666

[B7] Calvez S. L. Bourvon H. Kanaan H. Gatta S. M.-D. Philippot C. Reiss P. (2011). Enabling NIR imaging at room temperature using quantum dots, in Infrared Sensors, Devices, and Applications; and Single Photon Imaging II, (San Diego, CA: International Society for Optics and Photonics) 815506. 10.1117/12.893094

[B8] Chang A. Y. Liu W. Talapin D. V. Schaller R. D. (2014). Carrier dynamics in highly quantum-confined, colloidal indium antimonide nanocrystals. ACS Nano 8, 8513–8519. 10.1021/nn5031274 25106893

[B9] Chen M. Guyot-Sionnest P. (2017). Reversible electrochemistry of mercury chalcogenide colloidal quantum dot films. ACS Nano 11, 4165–4173. 10.1021/acsnano.7b01014 28314094

[B10] Chen M. Lu H. Abdelazim N. M. Zhu Y. Wang Z. Ren W.. (2017). Mercury telluride quantum dot based phototransistor enabling high-sensitivity room-temperature photodetection at 2000 nm. ACS Nano 11, 5614–5622. 10.1021/acsnano.7b00972 28525710

[B11] Chen M. Shao L. Kershaw S. V. Yu H. Wang J. Rogach A. L.. (2014). Photocurrent enhancement of HgTe quantum dot photodiodes by plasmonic gold nanorod structures. ACS Nano 8, 8208–8216. 10.1021/nn502510u 25020202

[B12] Chen M. Yu H. Kershaw S. V. Xu H. Gupta S. Hetsch F. (2013). Fast, air-stable infrared photodetectors based on spray-deposited aqueous HgTe quantum dots. Adv. Funct. Mater. 24, 53–59. 10.1002/adfm.201301006

[B13] Chernomordik B. D. Marshall A. R. Pach G. F. Luther J. M. Beard M. C. (2017). Quantum dot solar cell fabrication protocols. Chem. Mater. 29, 189–198. 10.1021/acs.chemmater.6b02939

[B14] Chuang C. H. Brown P. R. Bulović V. Bawendi M. G. (2014). Improved performance and stability in quantum dot solar cells through band alignment engineering. Nat. Mater. 13:796. 10.1038/nmat3984 24859641 PMC4110173

[B15] Chung D. S. Lee J.-S. Huang J. Nag A. Ithurria S. Talapin D. V. (2012). Low voltage, hysteresis free, and high mobility transistors from all-inorganic colloidal nanocrystals. Nano Lett. 12, 1813–1820. 10.1021/nl203949n 22385132

[B16] Coughlan C. Ibáñez M. Dobrozhan O. Singh A. Cabot A. Ryan K. M. (2017). Compound copper chalcogenide nanocrystals. Chem. Rev. 117, 5865–6109. 10.1021/acs.chemrev.6b00376 28394585

[B17] Cryer M. E. Halpert J. E. (2018). 300 nm spectral resolution in the mid-infrared with robust, high responsivity flexible colloidal quantum dot devices at room temperature. ACS Photon. 5, 3009–3015. 10.1021/acsphotonics.8b00738

[B18] De Iacovo A. Venettacci C. Colace L. Scopa L. Foglia S. (2017). Noise performance of PbS colloidal quantum dot photodetectors. Appl. Phys. Lett. 111:211104 10.1063/1.5005805

[B19] Della Gaspera E. Bersani M. Cittadini M. Guglielmi M. Pagani D. Noriega R.. (2013). Low-temperature processed Ga-doped ZnO coatings from colloidal inks. J. Am. Chem. Soc. 135, 3439–3448. 10.1021/ja307960z 23394063

[B20] Deng Z. Jeong K. S. Guyot-Sionnest P. (2014). Colloidal quantum dots intraband photodetectors. ACS Nano 8, 11707–11714. 10.1021/nn505092a 25343383

[B21] Dorfs D. Härtling T. Miszta K. Bigall N. C. Kim M. R. Genovese A. (2011). Reversible tunability of the near-infrared valence band plasmon resonance in cu2–xse nanocrystals. J. Am. Chem. Soc. 133, 11175–11180. 10.1021/ja2016284 21728384

[B22] Durmusoglu E. G. Yildizhan M. M. Gulgun M. A. Yagci Acar H. (2017). Production of small, stable PbS/CdS quantum dots via room temperature cation exchange followed by a low temperature annealing processes. J. Phys. Chem. C 121, 25520–25530. 10.1021/acs.jpcc.7b06153

[B23] Franke D. Harris D. K. Chen O. Bruns O. T. Carr J. A. Wilson M. W.. (2016). Continuous injection synthesis of indium arsenide quantum dots emissive in the short-wavelength infrared. Nat. Commun. 7:12749. 10.1038/ncomms12749 27834371 PMC5114595

[B24] Gao J. Nguyen S. C. Bronstein N. D. Alivisatos A. P. (2016). Solution-processed, high-speed, and high-quantum-efficiency quantum dot infrared photodetectors. ACS Photon. 3, 1217–1222. 10.1021/acsphotonics.6b00211

[B25] Gao J. Perkins C. L. Luther J. M. Hanna M. C. Chen H.-Y. Semonin O. E.. (2011). n-type transition metal oxide as a hole extraction layer in PbS quantum dot solar cells. Nano Lett. 11, 3263–3266. 10.1021/nl2015729 21688813

[B26] Geyer S. M. Scherer J. M. Jaworski F. B. Bawendi M. G. (2013). Multispectral imaging via luminescent down-shifting with colloidal quantum dots. Opt. Mater. Express 3, 1167–1175. 10.1364/OME.3.001167

[B27] Ghosh S. Saha M. De S. K. (2014). Tunable surface plasmon resonance and enhanced electrical conductivity of In doped ZnO colloidal nanocrystals. Nanoscale 6, 7039–7051. 10.1039/C3NR05608B 24842309

[B28] Goubet N. Jagtap A. Livache C. Martinez B. Portalès H. Xu X. Z.. (2018a). Terahertz HgTe nanocrystals: beyond confinement. J. Am. Chem. Soc. 140, 5033–5036. 10.1021/jacs.8b02039 29617124

[B29] Goubet N. Livache C. Martinez B. Xu X. Z. Ithurria S. Royer S.. (2018b). Wave-function engineering in HgSe/HgTe colloidal heterostructures to enhance mid-infrared photoconductive properties. Nano Lett. 18, 4590–4597. 10.1021/acs.nanolett.8b01861 29812951

[B30] Green M. Mirzai H. (2018). Synthetic routes to mercury chalcogenide quantum dots. J. Mater. Chem. C. 6, 5097–5112. 10.1039/C8TC00910D

[B31] Gresback R. Kramer N. J. Ding Y. Chen T. Kortshagen U. R. Nozaki T. (2014). Controlled doping of silicon nanocrystals investigated by solution-processed field effect transistors. ACS Nano 8, 5650–5656. 10.1021/nn500182b 24832958

[B32] Grigel V. Dupont D. De Nolf K. Hens Z. Tessier M. D. (2016). InAs colloidal quantum dots synthesis via aminopnictogen precursor chemistry. J. Am. Chem. Soc. 138, 13485–13488. 10.1021/jacs.6b07533 27701864

[B33] Guyot-Sionnest P. Roberts J. A. (2015). Background limited mid-infrared photodetection with photovoltaic HgTe colloidal quantum dots. Appl. Phys. Lett. 107:253104 10.1063/1.4938135

[B34] Hens Z. Moreels I. (2012). Light absorption by colloidal semiconductor quantum dots. J. Mater. Chem. 22, 10406–10415. 10.1039/C2JM30760J

[B35] Huo N. Gupta S. Konstantatos G. (2017). MoS2–HgTe quantum dot hybrid photodetectors beyond 2 μm. Adv. Mater. 29:1606576 10.1002/adma.201606576 28247438

[B36] Iacovo A. D. Venettacci C. Colace L. Scopa L. Foglia S. (2017). PbS colloidal quantum dot visible-blind photodetector for early indoor fire detection. IEEE Sens. J. 17, 4454–4459. 10.1109/JSEN.2017.2710301

[B37] Ihly R. Tolentino J. Liu Y. Gibbs M. Law M. (2011). The Photothermal Stability of PbS Quantum Dot Solids. ACS Nano 5, 8175–8186. 10.1021/nn2033117 21888407

[B38] Jagtap A. Goubet N. Livache C. Chu A. Martinez B. Gréboval C. (2018a). Short wave infrared devices based on HgTe nanocrystals with air stable performances. J. Phys. Chem. C 122, 14979–14985. 10.1021/acs.jpcc.8b03276

[B39] Jagtap A. Livache C. Martinez B. Qu J. Chu A. Gréboval C. (2018b). Emergence of intraband transitions in colloidal nanocrystals. Opt. Mater. Express 8, 1174–1183. 10.1364/OME.8.001174

[B40] Jean J. Xiao J. Nick R. Moody N. Nasilowski M. Bawendi M. (2018). Synthesis cost dictates the commercial viability of lead sulfide and perovskite quantum dot photovoltaics. Energy Environ. Sci. 11, 2295–2305. 10.1039/C8EE01348A

[B41] Jia B. W. Tan K. H. Loke W. K. Wicaksono S. Lee K. H. Yoon S. F. (2018). Monolithic integration of insb photodetector on silicon for mid-infrared silicon photonics. ACS Photon. 5, 1512–1520. 10.1021/acsphotonics.7b01546

[B42] Kanehara M. Koike H. Yoshinaga T. Teranishi T. (2009). Indium tin oxide nanoparticles with compositionally tunable surface plasmon resonance frequencies in the near-IR region. J. Am. Chem. Soc. 131, 17736–17737. 10.1021/ja9064415 19921844

[B43] Kershaw S. V. Susha A. S. Rogach A. L. (2013). Narrow bandgap colloidal metal chalcogenide quantum dots: synthetic methods, heterostructures, assemblies, electronic and infrared optical properties. Chem. Soc. Rev. 42, 3033–3087. 10.1039/C2CS35331H 23361653

[B44] Keuleyan S. Kohler J. Guyot-Sionnest P. (2014b). Photoluminescence of Mid-infrared HgTe colloidal quantum dots. J. Phys. Chem. C 118, 2749–2753. 10.1021/jp409061g

[B45] Keuleyan S. Lhuillier E. Brajuskovic V. Guyot-Sionnest P. (2011). Mid-infrared HgTe colloidal quantum dot photodetectors. Nat. Photonics 5, 489–493. 10.1038/nphoton.2011.142

[B46] Keuleyan S. E. Guyot-Sionnest P. Delerue C. Allan G. (2014a). Mercury telluride colloidal quantum dots: electronic structure, size-dependent spectra, and photocurrent detection up to 12 μm. ACS Nano 8, 8676–8682. 10.1021/nn503805h 25117471

[B47] Kim J. Choi D. Jeong K. S. (2018). Self-doped colloidal semiconductor nanocrystals with intraband transitions in steady state. Chem. Commun. 54, 8435–8445. 10.1039/C8CC02488J 29972153

[B48] Klem E. J. D. Gregory C. W. Temple D. S. Lewis J. S. (2015). Colloidal quantum dot Vis-SWIR imaging: demonstration of a focal plane array and camera prototype (Presentation Recording), in Optical Sensing, Imaging, and Photon Counting: Nanostructured Devices and Applications (San Diego, CA: International Society for Optics and Photonics), 955505 10.1117/12.2190372

[B49] Konstantatos G. Badioli M. Gaudreau L. Osmond J. Bernechea M. Garcia de Arquer F. P.. (2012). Hybrid graphene-quantum dot phototransistors with ultrahigh gain. Nat. Nanotechnol. 7, 363–368. 10.1038/nnano.2012.60 22562036

[B50] Kovalenko M. V. Kaufmann E. Pachinger D. Roither J. Huber M. Stangl J.. (2006). Colloidal HgTe nanocrystals with widely tunable narrow band gap energies: from telecommunications to molecular vibrations. J. Am. Chem. Soc. 128, 3516–3517. 10.1021/ja058440j 16536514

[B51] Kramer I. J. Levina L. Debnath R. Zhitomirsky D. Sargent E. H. (2011). Solar cells using quantum funnels. Nano Lett. 11, 3701–3706. 10.1021/nl201682h 21827197

[B52] Kriegel I. Jiang C. Rodríguez-Fernández J. Schaller R. D. Talapin D. V. da Como E.. (2012). Tuning the excitonic and plasmonic properties of copper chalcogenide nanocrystals. J. Am. Chem. Soc. 134, 1583–1590. 10.1021/ja207798q 22148506

[B53] Lai Y. Li H. Kim D. K. Diroll B. T. Murray C. B. Kagan C. R. (2014). Low-frequency (1/f) noise in nanocrystal field-effect transistors. ACS Nano 8, 9664–9672. 10.1021/nn504303b 25195975

[B54] Le-Van Q. Le Roux X. L. Aassime A. Degiron A. (2016). Electrically driven optical metamaterials. Nat. Commun. 7:12017. 10.1038/ncomms12017 27328976 PMC4917961

[B55] Lhuillier E. Guyot-Sionnest P. (2017). Recent progresses in mid infrared nanocrystal optoelectronics. IEEE J. Sel. Top. Quantum Electron. 23:6000208 10.1109/JSTQE.2017.2690838

[B56] Lhuillier E. Keuleyan S. Guyot-Sionnest P. (2012). Optical properties of HgTe colloidal quantum dots. Nanotechnology 23:175705. 10.1088/0957-4484/23/17/175705 22481378

[B57] Lhuillier E. Keuleyan S. Zolotavin P. Guyot-Sionnest P. (2013). Mid-infrared HgTe/As2S3 field effect transistors and photodetectors. Adv. Mater. 25, 137–141. 10.1002/adma.201203012 23027629

[B58] Lhuillier E. Pedetti S. Ithurria S. Heuclin H. Nadal B. Robin A.. (2014a). Electrolyte-gated field effect transistor to probe the surface defects and morphology in films of thick CdSe colloidal nanoplatelets. ACS Nano 8, 3813–3820. 10.1021/nn500538n 24601578

[B59] Lhuillier E. Robin A. Ithurria S. Aubin H. Dubertret B. (2014b). Electrolyte-gated colloidal nanoplatelets-based phototransistor and its use for bicolor detection. Nano Lett. 14, 2715–2719. 10.1021/nl5006383 24796385

[B60] Lhuillier E. Scarafagio M. Hease P. Nadal B. Aubin H. Xu X. Z.. (2016). Infrared photodetection based on colloidal quantum-dot films with high mobility and optical absorption up to THz. Nano Lett. 16, 1282–1286. 10.1021/acs.nanolett.5b04616 26753599

[B61] Liu H. Lhuillier E. Guyot-Sionnest P. (2014). 1/f noise in semiconductor and metal nanocrystal solids. J. Appl. Phys. 115:154309 10.1063/1.4871682

[B62] Liu Z. Janes L. M. Saniepay M. Beaulac R. (2018). Charge storage and quantum confinement resilience in colloidal indium nitride nanocrystals. Chem. Mater. 30, 5435–5443. 10.1021/acs.chemmater.8b02340

[B63] Livache C. Goubet N. Martinez B. Jagtap A. Qu J. Ithurria S.. (2018). Band edge dynamics and multiexciton generation in narrow band gap HgTe nanocrystals. ACS Appl. Mater. Interfaces 10, 11880–11887. 10.1021/acsami.8b00153 29578678

[B64] Livache C. Izquierdo E. Martinez B. Dufour M. Pierucci D. Keuleyan S.. (2017). Charge dynamics and optolectronic properties in HgTe colloidal quantum wells. Nano Lett. 17, 4067–4074. 10.1021/acs.nanolett.7b00683 28598629

[B65] Luther J. M. Jain P. K. Ewers T. Alivisatos A. P. (2011). Localized surface plasmon resonances arising from free carriers in doped quantum dots. Nat. Mater. 10, 361–366. 10.1038/nmat3004 21478881

[B66] Malinowski P. E. Georgitzikis E. Maes J. Vamvaka I. Frazzica F. Van Olmen J.. (2017). Thin-film quantum dot photodiode for monolithic infrared image sensors. Sensors 17:2867. 10.3390/s17122867 29232871 PMC5751686

[B67] Martinez B. Livache C. Goubet N. Jagtap A. Cruguel H. Ouerghi A. (2018). Probing charge carrier dynamics to unveil the role of surface ligands in HgTe narrow band gap nanocrystals. J. Phys. Chem. C 122, 859–865. 10.1021/acs.jpcc.7b09972

[B68] Martinez B. Livache C. Notemgnou Mouafo L. D. Goubet N. Keuleyan S. Cruguel H.. (2017). HgSe self-doped nanocrystals as a platform to investigate the effects of vanishing confinement. ACS Appl. Mater. Interfaces 9, 36173–36180. 10.1021/acsami.7b10665 28956432

[B69] Maurice A. Haro M. L. Hyot B. Reiss P. (2013). Quantum dots: synthesis of colloidal indium antimonide nanocrystals using stibine (Part. Part. Syst. Charact. 10/2013). Part. Part. Syst. Charact. 30, 821–821. 10.1002/ppsc.201370038

[B70] Melnychuk C. Guyot-Sionnest P. (2018). Slow Auger Relaxation in HgTe Colloidal Quantum Dots. J. Phys. Chem. Lett. 9, 2208–2211. 10.1021/acs.jpclett.8b00750 29648452

[B71] Park M. Choi D. Choi Y. Shin H. Jeong K. S. (2018). Mid-infrared intraband transition of metal excess colloidal Ag2Se nanocrystals. ACS Photon. 5, 1907–1911. 10.1021/acsphotonics.8b00291

[B72] Protière M. Nerambourg N. Renard O. Reiss P. (2011). Rational design of the gram-scale synthesis of nearly monodisperse semiconductor nanocrystals. Nanoscale Res. Lett. 6:472. 10.1186/1556-276X-6-472 21791060 PMC3211985

[B73] Qu J. Goubet N. Livache C. Martinez B. Amelot D. Gréboval C. (2018). Intraband mid-infrared transitions in Ag2Se nanocrystals: potential and limitations for Hg-free low-cost photodetection. J. Phys. Chem. C 122, 18161–18167. 10.1021/acs.jpcc.8b05699

[B74] Robin A. Livache C. Ithurria S. Lacaze E. Dubertret B. Lhuillier E. (2016). Surface control of doping in self-doped nanocrystals. ACS Appl. Mater. Interfaces 8, 27122–27128. 10.1021/acsami.6b09530 27640878

[B75] Rosencher E. (2002). Optoelectronics. Cambridge; New York, NY: Cambridge University Press.

[B76] Runnerstrom E. L. Bergerud A. Agrawal A. Johns R. W. Dahlman C. J. Singh A.. (2016). Defect engineering in plasmonic metal oxide nanocrystals. Nano Lett. 16, 3390–3398. 10.1021/acs.nanolett.6b01171 27111427

[B77] Sagar L. K. Walravens W. Maes J. Geiregat P. Hens Z. (2017). HgSe/CdE (E = S, Se) core/shell nanocrystals by colloidal atomic layer deposition. J. Phys. Chem. C 121, 13816–13822. 10.1021/acs.jpcc.7b02803

[B78] Sahu A. Khare A. Deng D. D. Norris D. J. (2012). Quantum confinement in silver selenide semiconductor nanocrystals. Chem. Commun. 48, 5458–5460. 10.1039/C2CC30539A 22540121

[B79] Sargent E. H. (2008). Solar cells, photodetectors, and optical sources from infrared colloidal quantum dots. Adv. Mater. 20, 3958–3964. 10.1002/adma.200801153

[B80] Sargent E. H. (2012). Colloidal quantum dot solar cells. Nat. Photon. 6, 133–135. 10.1038/nphoton.2012.33

[B81] Savich G. R. Pedrazzani J. R. Sidor D. E. Maimon S. Wicks G. W. (2011). Dark current filtering in unipolar barrier infrared detectors. Appl. Phys. Lett. 99:121112 10.1063/1.3643515

[B82] Savich G. R. Pedrazzani J. R. Sidor D. E. Wicks G. W. (2013). Benefits and limitations of unipolar barriers in infrared photodetectors. Infrared Phys. Technol. 59, 152–155. 10.1016/j.infrared.2012.12.031

[B83] Schimpf A. M. Lounis S. D. Runnerstrom E. L. Milliron D. J. Gamelin D. R. (2015). Redox chemistries and plasmon energies of photodoped In2O3 and Sn-doped In2O3 (ITO) nanocrystals. J. Am. Chem. Soc. 137, 518–524. 10.1021/ja5116953 25490191

[B84] Shen G. Guyot-Sionnest P. (2016). HgS and HgS/CdS colloidal quantum dots with infrared intraband transitions and emergence of a surface plasmon. J. Phys. Chem. C 120, 11744–11753. 10.1021/acs.jpcc.6b04014

[B85] Spencer B. F. Graham D. M. Hardman S. J. O. Seddon E. A. Cliffe M. J. Syres K. L. (2013). Time-resolved surface photovoltage measurements at $n$-type photovoltaic surfaces: Si(111) and ZnO(10$\backslash \bar{\{}1\}$0). Phys. Rev. B 88:195301 10.1103/PhysRevB.88.195301

[B86] Srivastava V. Dunietz E. Kamysbayev V. Anderson J. S. Talapin D. V. (2018). Monodisperse InAs quantum dots from aminoarsine precursors: understanding the role of reducing agent. Chem. Mater. 30, 3623–3627. 10.1021/acs.chemmater.8b01137

[B87] Sun Z. Liu Z. Li J. Tai G. A. Lau S.-P. Yan F. (2012). Infrared photodetectors based on CVD-grown graphene and PbS quantum dots with ultrahigh responsivity. Adv. Mater. 24, 5878–5883. 10.1002/adma.201202220 22936561

[B88] Talapin D. V. Murray C. B. (2005). PbSe nanocrystal solids for n- and p-channel thin film field-effect transistors. Science 310, 86–89. 10.1126/science.1116703 16210533

[B89] Tan L. Li P. Sun B. Chaker M. Ma D. (2017). Stabilities related to near-infrared quantum dot-based solar cells: the role of surface engineering. ACS Energy Lett. 2, 1573–1585. 10.1021/acsenergylett.7b00194

[B90] Tandon B. Yadav A. Khurana D. Reddy P. Santra P. K. Nag A. (2017). Size-induced enhancement of carrier density, LSPR quality factor, and carrier mobility in Cr–Sn doped In2O3 Nanocrystals. Chem. Mater. 29, 9360–9368. 10.1021/acs.chemmater.7b03351

[B91] Tang X. Ackerman M. M. Guyot-Sionnest P. (2018). Thermal imaging with plasmon resonance enhanced HgTe colloidal quantum dot photovoltaic devices. ACS Nano 12, 7362–7370. 10.1021/acsnano.8b03871 29985583

[B92] Tang X. Tang X. Lai K. W. C. (2016). Scalable fabrication of infrared detectors with multispectral photoresponse based on patterned colloidal quantum dot films. ACS Photonics 3, 2396–2404. 10.1021/acsphotonics.6b00620

[B93] Wang H. Lhuillier E. Yu Q. Mottaghizadeh A. Ulysse C. Zimmers A. (2015). Effects of electron-phonon interactions on the electron tunneling spectrum of PbS quantum dots. Phys. Rev. B 92:041403 10.1103/PhysRevB.92.041403

[B94] White A. M. (1987). Infra Red Detectors. Available online at: https://patents.google.com/patent/US4679063A/en (Accessed September 13, 2018)

[B95] Yakunin S. Dirin D. N. Protesescu L. Sytnyk M. Tollabimazraehno S. Humer M.. (2014). High infrared photoconductivity in films of arsenic-sulfide-encapsulated lead-sulfide nanocrystals. ACS Nano 8, 12883–12894. 10.1021/nn5067478 25470412 PMC4278417

[B96] Yifat Y. Ackerman M. Guyot-Sionnest P. (2017). Mid-IR colloidal quantum dot detectors enhanced by optical nano-antennas. Appl. Phys. Lett. 110:041106 10.1063/1.4975058

[B97] Zhang H. Zhang R. Schramke K. S. Bedford N. M. Hunter K. Kortshagen U. R. (2017). Doped silicon nanocrystal plasmonics. ACS Photonics 4, 963–970. 10.1021/acsphotonics.7b00026

